# Prescriber and Patient Responsibilities in Treatment of Acute Respiratory Tract Infections — Essential for Conservation of Antibiotics

**DOI:** 10.3390/antibiotics2020316

**Published:** 2013-06-04

**Authors:** Alike van der Velden, Martin G. Duerden, John Bell, John S. Oxford, Attila Altiner, Roman Kozlov, Aurelio Sessa, Antonio C. Pignatari, Sabiha Y. Essack

**Affiliations:** 1 Julius Center for Health Sciences and Primary Care, University Medical Center Utrecht, Heidelberglaan 100, 3584 CX, Utrecht, The Netherlands; 2 Office of Medical Director BCUHB, PO Box 18, Croesnewydd Road, Wrexham LL13 7TD, UK; E-Mail: martin@theduerdens.co.uk; 3 Pharmacy School, University of Technology Sydney, Broadway 2007, Australia; E-Mail: john.bell@nexonline.com.au; 4 Centre for Infectious Diseases, Bart’s and the London Queen Mary’s School of Medicine and Dentistry, Retroscreen Virology Ltd., 327 Mile End Road, London E1 4NS, UK; E-Mail: j.oxford@retroscreen.com; 5 Institute of General Practice, Rostock University Medical Centre, Rostock 18051, Germany; E-Mail: altiner@med.uni-rostock.de; 6 Institute of Antimicrobial Chemotherapy, Smolensk State Medical Academy, 28 Krupskaya Str, Smolensk 214019, Russia; E-Mail: roman.kozlov@antibiotic.ru; 7 Italian College of General Practitioners, Florence 50142, Italy; E-Mail: sessa.aurelio@simg.it; 8 Escola Paulista de Medicina-Universidade Federal de São Paulo, Rua Leandro Dupret 188, Sao Paulo, SP 04025-010, Brazil; E-Mail: pignatari@terra.com.br; 9 School of Health Sciences, University of KwaZulu-Natal, Private Bag X54001, Durban 4000, South Africa; E-Mail: essacks@ukzn.ac.za

**Keywords:** antibiotics, communication, primary care, resistance, symptomatic management, respiratory tract infection

## Abstract

Inappropriate antibiotic use in normally self-limiting acute respiratory tract infections (RTIs), such as sore throat and the common cold, is a global problem and an important factor for increasing levels of antibiotic resistance. A new group of international experts—the Global Respiratory Infection Partnership (GRIP)—is committed to addressing this issue, with the interface between primary care practitioners and their patients as their core focus. To combat the overuse of antibiotics in the community, and facilitate a change from prescribing empiric antibiotic treatment towards cautious deferment combined with symptomatic relief, there is a need to introduce and enhance evidence-based dialogue between primary care practitioners and their patients. Communication with patients should focus on the de-medicalisation of self-limiting viral infections, which can be achieved via a coherent globally endorsed framework outlining the rationale for appropriate antibiotic use in acute RTIs in the context of antibiotic stewardship and conservancy. The planned framework is intended to be adaptable at a country level to reflect local behaviours, cultures and healthcare systems, and has the potential to serve as a model for change in other therapeutic areas.

## 1. Introduction

Antibiotic resistance exerts a significant and pressing global healthcare challenge, with 25,000 people in Europe dying each year as a direct result of resistant infection [1]. Using 2007 data, the costs of related healthcare expenditure and productivity loss for the European Union member states, Iceland and Norway, were estimated to be as high as 1.5 billion Euros per year [1]. In America, hospital-acquired infections, most of which are caused by antibacterial-resistant pathogens, result in an estimated 99,000 deaths per year [2]. Gram-negative bacteria, in particular, become resistant to antibiotics, resulting in, for example, carbapenem-resistant *Enterobacteriaceae* spp. and extended-spectrum beta-lactamase-producing *Enterobacteriaceae* [3,4,5,6]. Certain gram-negative pathogens are now panresistant, where none of the currently available antibiotics are effective [6,7,8]. In South Asia, antimicrobial resistance is estimated to cause 300,000 infant deaths annually from neonatal sepsis-causing organisms, such as *Staphylococcus aureus*,* Escherichia coli*,* Enterobacter* and *Acinetobacter* species and *Pseudomonas* [9]. The problem is thought to be equally widespread, yet under-investigated, and consequently under-estimated and under-reported, in other developing countries.

While antibiotic resistance has predominantly been a clinical problem in hospital settings, recent data show resistant organisms have also been detected in patients outside of this setting [10], with higher resistance rates in countries with high levels of antibiotic use [11]. Diseases associated with antimicrobial resistance outside the hospital setting include tuberculosis, gonorrhoea (specifically *Neisseria* *gonorrhoeae*), typhoid fever and Group B *streptococcus* [12]. Community-acquired antimicrobial resistance is of particular concern as these infections can be common and easily transmitted [10]. Infection with antibiotic-resistant bacteria may cause severe illness, increased mortality rates, an increased risk of complications and admission to hospital, often resulting in higher treatment costs [13,14,15]. 

Some recent emerging trends that are of growing importance should also be considered. One is the rise in prescribing for urinary tract infections. Even in countries such as the Netherlands, which has a low and stable antibiotic prescribing rate, a 32% increase in prescribing for these infections was noted between 2007–2011 [16]. In other European countries increased resistance to the standard urinary tract infection treatment, nitrofurantoin, is also developing [17]. This is being driven by increasing antimicrobial resistance of *Enterobacteriaceae*, such as *Escherichia coli* and *Klebsiella* *pneumoniae* [18]. This poses a significant public health problem, with therapeutic options to treat life-threatening *K. pneumoniae* infections diminishing [18]. 

In the context of few innovative or new antibiotics in the drug development pipeline, the World Health Organization (WHO) describes a future of a post-antibiotic world and warns that not only will this eliminate the advances in healthcare made over the past 100 years, that have ensured longer life in most parts of the developed and developing worlds, but it may also result in simple infections becoming unmanageable and potentially fatal [19,20]. The limited use of antibiotics in the future does not appear to be sufficient enough to change behaviour in antibiotic prescribing and self-initiated antibiotic use. The challenge facing societies around the globe is to encourage significant changes now to save lives in the future.

GRIP is a new multi-disciplinary group of international experts, comprising of primary care and hospital physicians, microbiologists, researchers and pharmacists. GRIP members were selected due to their existing interest and work in the field of antibiotic use and resistance. In particular, all members are focused on implementing change in antibiotic use and the treatment of non-serious infections, such as respiratory tract infections (RTIs), in primary care. The need for a multidisciplinary group of experts with broad reach was also considered when identifying candidates, to maximise the impact GRIP is intending to have as a campaigning force for change. GRIP is committed to address antibiotic overuse-related problems using evidence-based studies to support suitable rationale for antibiotic use and promote antibiotic stewardship among healthcare professionals. A framework is being developed for non-antibiotic treatment options for symptoms of acute RTIs.

In this first GRIP opinion paper, the group aims to stimulate debate between healthcare professionals and highlight the need for an integrated approach to facilitating behavioural change among antibiotic prescribers and users.

A pilot for the development of such an integrated approach should be a single infection, the treatment of which is currently characterised by the overuse of antibiotics in the primary care sector—an ineffective and unnecessary treatment option for the majority of patients—and where alternative symptomatic relief options are available. GRIP identified that the majority of patients who develop acute RTIs, such as sore throat and common colds (with the exception of the more severe infections such as pneumonia), can adopt this approach. Once a coherent international approach has been developed to promote a change in the prescribing and use of antibiotics for the treatment of RTIs, this could serve as a model for change in other infections.

## 2. Discussion

### 2.1. Resistance and Antibiotic Use

Data show a direct correlation between the use of antibiotics and resistance. Countries where there is higher consumption of antibiotics show higher resistance rates [11,21]. The converse has also been shown, with Finnish data finding macrolide resistance of *S. pyogenes* reducing in parallel with a decrease in antibiotic consumption. Antibiotic resistance dropped from 9.2% in 1997 to 7.4% in 2000, with a statistically significant association between regional macrolide resistance and consumption rates [22].

Taking Europe as an example, antibiotic usage varies widely, as do resistance rates. The most recent data from the European Antibiotic Surveillance Reports found that antibiotic resistance rates of *E. coli* and/or *K. pneumoniae* varied markedly between countries. Resistance of both were among the highest in Greece and Slovakia and the lowest in Sweden and Norway in 2011 [18]. Rates of resistant *E. coli* varied by a factor of 18 between Sweden (1.0%) and Greece (18.2%); for *K. pneumoniae* the differences were even more pronounced, ranging from 0.7% in Sweden to 64.1% in Greece [18]. This correlates to antibiotic consumption levels. On average, the European consumption rate for antibiotics is 18.3 defined daily doses (DDD)/1000 inhabitants/day in 2010, with a low rate seen in Sweden (~14 DDD/inhabitants/day) and the highest in Greece (~39 DDD/inhabitants/day), France and Luxembourg (both ~28 DDD/inhabitants/day) [23].

The impact of high antibiotic use on antibiotic resistance is significant [24]. Global data from 2010/2011 for the percentage of extended spectrum beta-lactamase [ESBL]-producing bacteria present, indicating antibiotic resistance of *E. coli* and *Klebsiella* spp., are highest in India and China at ≥80% and ≥60%, respectively [13]. Rates were ≤30% in Latin America, East/Southeast Asia and Southern Europe, and 5%–10% in Northern Europe, Australasia and North America [13]. European data from 2011 demonstrate an alarming increase in resistance of these organisms, with around a third of European countries showing a rise in combined resistance to third-generation cephalosporins, fluoroquinolones and aminoglycosides over the previous four years [18].

This will exert a major adverse effect on treatment options. A recent review describing patients with urinary tract and respiratory tract bacterial infections treated with antibiotics reported that individual resistance may persist for up to 12-months post-treatment, thereby creating situations for the need of second-line antibiotics [25]. When antibiotics are used the individual’s entire bacterial gene pool is sensitive to change. As resistance can be transferred between bacteria, new pathogens can evolve. Therefore, antibiotic use in an individual could have a direct impact on the level of resistance in the population [25].

In developing countries, the impact of rapidly increasing resistance rates is of particular importance and concern. Respiratory disease has overtaken diarrhoea as the most frequent cause of child death in these countries, [26] with community-acquired *S. pneumoniae* among the main pathogenic species. This organism also demonstrates increasing resistance to a variety of antibiotic agents [27]. 

### 2.2. Changing the Dialogue between Healthcare Professionals and Patients

Misconceptions and uncertainties regarding the role of antibiotics exist among patients and primary care physicians [28]. For example, a survey in Australia revealed that only 50% of patients are conscious of the development of antibiotic resistance, with even fewer (40%) aware that antibiotics are ineffective against viruses [29]. This echoes European research reporting that around half of patients believed antibiotics were effective in treating viruses, cold and flu [30].

RTIs are the most commonly treated acute problem in primary care [31], with the majority of infections of viral origin. In patients with RTIs at high risk of developing complications the use of antibiotics needs to be considered. However, complication rates have markedly reduced [31,32] and the need for antibiotics is no longer required in the majority of people with RTIs. Furthermore, in developed countries with comparatively low antibiotic use, complications are no more frequent than in developed countries with high antibiotic use.

Despite this, antibiotic use for RTIs in primary care remains high. In Europe, upper RTIs accounted for 57% of antibiotics used, with a further 30% for lower RTIs; in contrast the next most common condition was urinary tract infections at 7% [30]. In the UK, 60% of all antibiotics prescribed are for patients with RTIs [33]. In Bangkok, Thailand, the prevalence of antibiotic prescription for some RTIs was as high as 78%–94% [34]. A lower prescription rate of 33.5% was observed in Norway for patients with RTIs seen in primary care [35]. In Italy, antibiotics were prescribed for 44% of croup, influenza and common cold cases presenting in primary care [36]. 

There also appears to be a dissonance between physician and patient expectations during a RTI consultation. Primary care physicians say they often feel under pressure by the patient to prescribe antibiotics [37]. Some studies report that patients strongly influence the antibiotic prescribing of physicians by using a number of different behaviours, such as “in the next two days I must leave for my holidays” or “I got antibiotics for this before and it worked” [38]. Other studies report that only a small proportion of patients explicitly expect a prescription [39], and even if they do, their primary concern is to find symptomatic relief [37,40]. In a Dutch survey, patients who, prior to the consultation indicated a desire for an antibiotic, were equally satisfied with the consultation afterwards, irrespective of whether they received an antibiotic or not [41]. Furthermore, a clear explanation about the expected course and duration of disease, and a proper physical examination correlated more to patient satisfaction than a prescription.

Improved communication in primary care can help bridge this gap between physician and patient expectations. This can be achieved using various approaches. In Germany, training physicians in patient-centered communication resulted in a 40% reduction in antibiotic prescribing, with results lasting for over one year [28]. In comparison, the control group of doctors with no training in communication skills increased their antibiotic prescribing during the study period. In the UK, the paediatric antibiotic prescribing rate decreased by 50% as a result of physicians using an interactive information leaflet on the management of upper RTIs in children during the consultation with parents [31]. The limited use of diagnostic techniques in general practice to identify the aetiological agent (viral or bacterial) might also explain the overuse of antibiotic therapy. In fact, where diagnostic rapid tests are used there is a lower risk of antibiotic use [36]. However, as the majority of infections, bacterial or viral, are self-limiting and symptomatic relief is adequate, GRIP raises the issue of the need for testing. 

### 2.3. A Global Framework for the Non-Antibiotic Treatment of RTIs

There are various national and global initiatives to try to counteract the threat of antibiotic resistance. In 2010, the Infectious Diseases Society of America (IDSA) started a new campaign, the “10×'20” initiative, which is a global commitment to support the development of ten novel, effective antibiotics by 2020 [42]. An update in 2013 on the incentive highlighted the slow progression in the clinical development of new antibacterial agents for resistant gram-negative bacteria [43]. In addition to the development of new antimicrobial agents, IDSA called for more focus on antibiotic stewardship and infection prevention in order to preserve the efficacy of current antibiotics [43]. The UK government published a report in March 2013 identifying antimicrobial resistance as an area requiring profound action due to the impact on global health [43]. Emphasis was placed on the preservation of antibiotics and tips were provided on effective antibiotic prescribing. The report stated: “vital to improving antimicrobial stewardship will be the education, training and workforce priorities of healthcare and public health professionals” and a detailed UK 2013–2018 antimicrobial resistance strategy and action plan will be published in 2013 [44].

In many countries there are education campaigns that aim to change healthcare professional and patient behaviour on antibiotic consumption. These include initiatives from the US Center for Disease Control and Prevention, first launched in 1995, and the European Centre for Disease Prevention and Control that has been running a European-wide antibiotic awareness day since 2008. In addition, many countries have run their own national awareness measures, such as Australia (‘Resistance fighters’), France (‘Antibiotics, not automatic’, underway since 2001), Germany (the DART—Deutsche Antibiotika-Resistenzstrategie) and Thailand (‘Antibiotics Smart Use’, running since 2007).

The impact of these campaigns was diverse. An analysis of 22 national or regional level campaigns in high-income countries did find a reduction in antibiotic use. As all but one campaign targeted the patient and healthcare professional simultaneously [45], it cannot be concluded whether patient education and awareness alone is an effective intervention to decrease antibiotic use. 

Yet, despite an 18-year awareness campaign in the US, antibiotic overprescribing for paediatric patients remains high at 60%, and research on a UK awareness campaign also found little impact. Polish data suggest that a third of the population changes behaviour due to the European Antibiotic Awareness day; prescribing rates however remain high [46].

It is unclear from current study data whether these effects are sustainable. In France, the awareness initiative reduced antibiotic prescribing by 26.5% in the first five years [47]. A more recent survey, however, showed that 42% of patients had taken antibiotics in the previous year, of which a third were for upper RTIs [30]. It is also difficult to determine what is more effective: changing patient knowledge and perception of antibiotics or physician prescribing behaviour. European research showed that advice from a doctor and television advertising are the most effective means of educating patients on appropriate antibiotic use [30]. As the latter is often cost-prohibitive in many countries, the focus for change needs to be on changing doctor and supplementary healthcare professional behaviour. In a research context, this has been shown to work. An analysis of interventions to improve antibiotic prescribing for RTIs among primary care physicians resulted in, on average, an 11.6% reduction in antibiotic prescribing [48].

Despite the range of campaigns and initiatives, educating healthcare professionals, and also training them how to educate patients on antibiotic use is essential. More could be done to optimise the interaction between patient and healthcare professionals during the consultation for an acute RTI. Numerous regional, national and international guidelines are in place outlining appropriate prescribing and many educational initiatives exist that focus on raising awareness of antibiotic resistance globally. GRIP believes that this is where the focus in countering antibiotic resistance should lie and calls for the development of an international framework for the non-antibiotic management of RTIs that specifically addresses the physician-patient interaction.

In addition to setting out the rationale for appropriate antibiotic use in RTIs and outlining non-antibiotic management strategies, the framework should cover several key topics (Table 1). 

**Table 1:** Framework outline for the non-antibiotic treatment of acute respiratory tract infections.

Policy to advance antibiotic stewardship and conservancy imperatives Prevention of inappropriate antibiotic use by providing guidance on the indications, signs and symptoms of RTIs, and subgroups of patients where antibiotic use is appropriate Patient participation to encourage patient empowerment, combined with appropriate evidence-based symptomatic management of RTIs Prescriber guidance on strategies for an effective dialogue between primary care healthcare professionals and patients during and after consultation for a RTI, resulting in clear take-home messages, supplemented with appropriate materials or referral to available resources, including, but not limited to: acknowledging the reasons for patient consultation for an RTI providing reassurance and counselling on non-antibiotic treatment, resulting in patient satisfaction with the consultation offering evidence-based, symptom-focused advice educating the patient on antibiotic conservancy outlining what follow-up is required and what symptoms would necessitate further intervention

RTI: Respiratory tract infection.

The development of this framework should be cognisant of effective behavioural change initiatives from various countries and involve multiple stakeholders. The framework has to be robust enough to set a benchmark for care, while allowing adaptation at country level to reflect local realities. For example, in some countries the enforcement of legal or fiscal measures would be of great value to restrict inappropriate antibiotic sales through pharmacies and to incentivise primary care physicians to reduce prescribing.

GRIP is working to further develop this framework, which will be supplemented by practical materials to educate primary care healthcare professionals, pharmacists and patients in changing behaviour and perceptions around antibiotic use in RTI treatment, and to encourage symptomatic relief. A key component of this will be guidance for healthcare professionals on having a constructive dialogue with patients that allows patients’ needs for symptom relief to be met without the need for an antibiotic prescription. Materials will be available through a GRIP website.

A summit meeting is to be held in the summer of 2013 to convene antibiotic resistance expertise from around the globe in order to finalise the framework into one that facilitates prudent antibiotic use via targeted, nationwide actions. Representatives will span both the developing world (Brazil, China, India, Malaysia, Russia, South Africa, Singapore and Thailand) and developed countries (Australia, Austria, Czech Republic, Germany, Ireland, Israel, Italy, Middle East, Netherlands, Switzerland, the United Kingdom and the United States of America). 

The core GRIP group also aims to include members from developing countries where antibiotic resistance poses particular problems, such as India and Thailand. 

## 3. Conclusions

Inappropriate antibiotic use in normally self-limiting RTIs is common in many countries and is contributing to the increase in antibiotic resistance. To reverse this tendency, a multi-faceted international, collaborative framework needs to be developed that facilitates behavioural change towards a non-antibiotic, patient-centered symptomatic management strategy in primary care. This framework should not only set out the rationale for why appropriate antibiotic use in RTIs is essential, but it should particularly outline how to enforce its implementation to change practice through improved dialogue between the healthcare provider and patient, as articulated in the framework outline. The framework should be adaptable at country level to reflect cultural sensitivities, differing healthcare provision systems and national guidelines, and could serve as a model for change in other therapeutic sectors where overuse of antibiotics in the primary care is of concern. This framework will be supplemented with practical materials that facilitate conversations between healthcare professionals and patients to promote appropriate antibiotic use.
